# Effect of short-term psychological intervention on anxiety of pregnant women with positive screening results for chromosomal disorders: a randomized controlled trial

**DOI:** 10.1186/s12884-021-04206-5

**Published:** 2021-11-09

**Authors:** Arezoo Bayat, Leila Amiri-Farahani, Mehdi Soleimani, Nooshin Eshraghi, Shima Haghani

**Affiliations:** 1 Department of Reproductive Health and Midwifery, Shahid Akbarabadi Clinical Research Development Unit (Sh ACRDU), School of Nursing and Midwifery, Iran University of Medical Sciences, Tehran, Iran; 2 Department of Reproductive Health and Midwifery, Nursing Care Research Center, School of Nursing and Midwifery, Iran University of Medical Sciences, Tehran, 1996713883 Iran; 3 Department of Psychiatry, School of Medicine, Tehran University of Medical Sciences, Tehran, Iran; 4 Department of Obstetrics and Gynecology, Iran University of Medical Sciences, Akbarabadi Teaching Hospital, Tehran, Iran; 5 Department of Biostatistics, Nursing Care Research Center, Iran University of Medical Sciences, Tehran, Iran

**Keywords:** Anxiety, Pregnancy screening, Group intervention, Individual intervention, Cognitive-behavioral training

## Abstract

**Background and aim:**

Prenatal diagnosis of fetal abnormalities is a critical and stressful event for women. Most pregnant women are concerned about fetal abnormalities and screening tests. Due to the importance of anxiety reduction in pregnant women, this study was conducted to determine the effect of short-term psychological intervention on the anxiety of pregnant women with positive screening results for chromosomal disorders.

**Methods:**

A randomized clinical trial was performed on women referred to Akbarabadi Hospital in Tehran, Iran, who had positive screening results for chromosomal abnormalities. Participants were selected from eligible individuals by a continuous method and were assigned to two groups of cognitive-behavioral training (*n* = 46) and control (*n* = 46), using the block balanced randomization method. Participants in the cognitive-behavioral training group received 4 sessions of individual counseling. The control group received routine pregnancy visits. The Spielberger State-Trait Anxiety Inventory was completed before the intervention and immediately at the end of the intervention (before receiving the amniocentesis result). The analysis of intervention effects was performed as intention-to-treat and per-protocol analysis.

**Results:**

There was a statistically significant difference in post-intervention state anxiety scores and trait anxiety scores (*p* <  0.001) between the intervention and control groups, when their means were adjusted for pre-intervention scores for both intention-to-treat and per-protocol analysis. Also, there was a large effect size between the groups in terms of state (ITT: η_p_^2^ = 0.63, PP: η_p_^2^ = 0.71) and trait (ITT: η_p_^2^ = 0.72, PP: η_p_^2^ = 0.75) anxiety scores clinically for both intention-to-treat and per-protocol analysis. The intervention group had a statistically significant and large decrease in state and trait anxiety scores from pretrial to post-trial. In contrast, the control group had a statistically significant and medium increase in state and trait anxiety scores from pretrial to post-trial.

**Conclusion:**

The results showed that cognitive-behavioral training reduced the anxiety of pregnant women with positive screening results for chromosomal disorders. According to the results, it is recommended to hold cognitive-behavioral training classes to reduce the anxiety of pregnant women with a positive screening result for chromosomal disorders.

**Trial registration:**

IRCT.ir: IRCT20180427039436N7; date of registration: 24/08/2020 2020-08-24.

**Supplementary Information:**

The online version contains supplementary material available at 10.1186/s12884-021-04206-5.

## Introduction

Pregnancy is an important stage in women’s life, and confronts them with the growth of another human being inside their body, which is associated with many psychological, emotional, and physical pressures [1]. It also increases the vulnerability and anxiety of women [2]. Pregnancy and the year after birth have been reported as particularly vulnerable times for the onset or recurrence of anxiety disorders in women [3].

According to the results of a meta-analysis by Dennis et al., the prevalence of anxiety in the first, second and third trimester is 19.2, 18.1, and 24.6%, respectively [4]. In this regard, studies in Iran have assessed pregnancy-related anxiety by general anxiety questionnaires and reported prevalence rates of 32.5 and 40% [5, 6]. Severe maternal anxiety can negatively affect women’s physical and mental health and their children’s cognitive, emotional, and behavioral development [7]. Severe and prolonged anxiety increases the likelihood of preterm labor [8].

The most common causes of anxiety include fears of dying during childbirth, labor pain, loss of baby, and fetal abnormalities, and also the baby’s health has been reported to be the most common concern of mothers [9]. Most pregnant women are concerned about fetal abnormalities and screening tests [10].

Prenatal screening is recommended for pregnant women as part of routine prenatal care in many countries [11]. Prenatal screening is routinely offered to many pregnant women in the first and second trimester to look for birth defects. In the first trimester, between 11 weeks to 13 weeks + 6 days of pregnancy, a combined screening test is performed, which measures the thickness of nuchal translucency (NT) and maternal serum markers such as pregnancy-associated plasma protein-A (PAPP-A) and free-beta human chorionic gonadotropin (Free β-hCG) [12–14]. In the second trimester, the maternal serum screening is performed around 15-22 weeks of gestation. These blood tests investigate abnormal levels of proteins and hormones such as alpha-fetoprotein (AFP), unconjugated estriol (E3), human chorionic gonadotropin (hCG), and inhibin-A [14, 15]. Results of a study in Kermanshah, Iran revealed that among the women screened in the first and second trimester, 1.22 and 4.13% of pregnancies were screen-positive for Down’s syndrome, respectively [16].

Screening tests, invasive tests, and long waiting times for the test results increase the anxiety of pregnant women [17, 18]. A study showed that anxiety scores were higher in pregnant women who had positive screening tests [19]. In another study, anxiety and depression scores in the group that underwent amniocentesis were significantly higher than the control group [20]. In addition to careful screening tests, their psychological aspects should also be considered and intervention methods such as counseling should be used to reduce stress in pregnant women [21].

A systematic review study that was conducted to evaluate the effects of non-pharmacological interventions on reducing stress and anxiety of pregnant women showed that there is a need for quality and sufficient clinical trials to evaluate interventions that aim to deal with psychological disorders in pregnant women [22]. In another systematic review, the results of a meta-analysis on three mindfulness studies showed that mindfulness intervention has no effect on reducing pregnancy anxiety, and also meta-analysis was not possible in other types of interventions due to the small number of clinical trial studies and heterogeneity of interventions. Therefore, it can be argued that there is insufficient evidence to draw general conclusions about the benefits of psychological interventions in reducing pregnancy anxiety [23]. Despite the evidence supporting the efficacy of cognitive-behavioral therapy (CBT) in the treatment of anxiety disorders in the general population, few studies have specifically examined the effect of CBT on the treatment of prenatal anxiety [24]. The results of a study aimed at evaluating the effectiveness of CBT on prenatal anxiety showed that participants in the intervention group had a significant reduction in prenatal anxiety [25]. In another study, internet CBT had a negative effect on gestational depression, with some participants reporting concern about continuing and pursuing treatment programs, and some having lower depression scores [26].

Screening is one of the new care methods in pregnancy and performing screening tests and their results cause anxiety in women. On the other hand, systematic review studies, meta-analyses, and clinical trials have produced contradictory results regarding the effectiveness of interventions in reducing pregnancy anxiety. Therefore, this study was conducted to determine the effect of short-term psychological intervention based on cognitive-behavioral training on the anxiety of pregnant women with positive screening results for chromosomal disorders.

## Methods

### Trial design and participants

This study is a parallel randomized clinical trial with intervention and control groups. Reporting of this study is in accordance with the Consolidation Standards of Reporting Trials (CONSORT) statement (Additional file 1), [27]. This study has been funded by Iran University of Medical Sciences. The study’s protocol was registered in the Clinical Trial Registration Center on August 24, 2020 with the code: IRCT20180427039436N7. The study population consisted of women, who had positive screening results for chromosomal disorders in the first stage. They had been referred to the perinatology clinic of Shahid Akbrabadi Hospital in Tehran, Iran for amniocentesis.

Inclusion criteria were; being Iranian, being Persian-speaking, having gestational age of 11-15 weeks, being a nulliparous woman, having a singleton pregnancy, having state anxiety score of between 31 and 75, and trait anxiety score of between 31 and 72 [28–30], and having at least a high school diploma. Exclusion criteria were; having a history of substance abuse, infertility, and serious psychiatric disorders including psychotic disorders, bipolar disorder, and depression, and having any identified psychological disorders that require medication. Withdrawal criteria included; not attending more than one training session, unwillingness to continue participating in the study, having abortion during the study, and occurrence of unfortunate events such as the death of loved ones during the training sessions.

In order to collect data, the researcher first selected eligible individuals among women, who had positive screening results for chromosomal disorders in the first stage, by convenience sampling and provided them with the necessary explanations on the study process. Then, those who agreed to take part in the study were enrolled in the study. The recruitment of pregnant women lasted for about 3 months. The intervention began in September 2020, and the follow-up ended in December 2020. The eligible women were assigned to two groups of intervention and control using the block randomization method at a ratio of 1:1 (available at http://www.randomization.com). To determine the sequence of participants’ allocation based on the block balanced randomization method, it is necessary to know about the total sample size, the number of groups, and the number of group repetitions in each block (which was considered equal). In the current study, the size of each block was twice as big as the number of groups (4 groups in each block). An epidemiologist, who was not part of the study, made a randomization list. For allocation concealment, the assignment list remained with the epidemiologist. Blinding was not possible in this trial due to the nature of the interventions. The tool, which was completed by the participant before and after the study, was provided by the researcher’s assistant as an outcome assessor and she did not know the study groups and their allocation. Also, statistical analysis was performed by a statistician who also did not know the content provided for the study groups and their allocation.

**Fig. 1:**
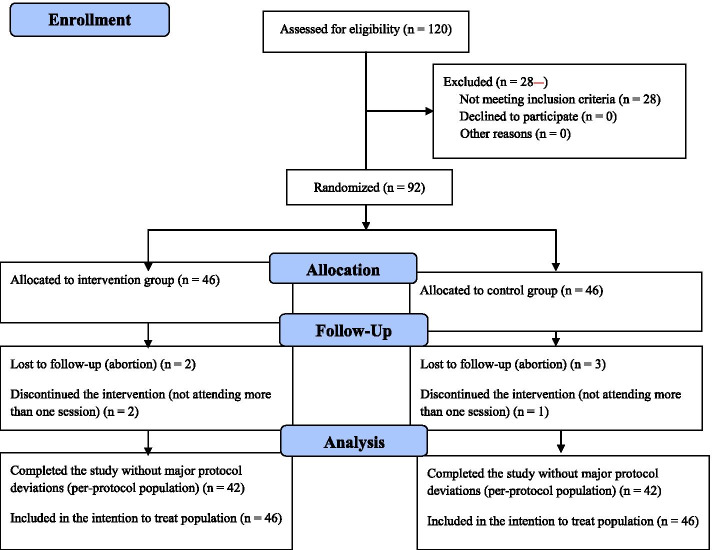
Consort flow diagram of study

Figure 1 shows the process of participants’ entry and exit during the clinical trial.

A total of 92 samples were included in the study. In the intervention group, 4 people were excluded (2 due to abortion during the study and 2 due to participating in only one session). Also in the control group, 4 people were excluded (3 due to abortion during the study and 1 for unwillingness to continue with the study).

### Description of intervention

The content of cognitive-behavioral training in the intervention group was derived from the practical guide to group cognitive therapy by Michael Frey [31], and Rena Banche & Rob Wilson cognitive-behavioral therapy [32]. A total of four 45 to 60-min sessions were held individually twice a week by the researcher. The content of the training session is given in Table 1. The sessions were conducted by a certified midwife in a cognitive-behavioral training under the supervision of a clinical psychologist. The control group only participated in the routine prenatal classes. It should be noted that the content of cognitive-behavioral training was provided to the control group as a training booklet after the sampling. Participants in both groups were followed up until the implementation of amniocentesis.

**Table 1:** Intervention group training program (cognitive-behavioral training)

Session	Structure and content of meetings	Practice in each session and homework
First session	Introduction of the researcher, overview of the content of sessions, expression of clients’ feelings about the result of screening, training about the importance, features, benefits, risks, and differences between screening tests in the first and second trimester, and also anxiety and relaxation training.	Making a chart at home and writing down all negative thoughts and the degree of belief in them, and also the negative emotions and their intensity.
Second session	Review of previous session homework and misconceptions about the cause of screening anxiety, A-B-C chain training (A for activating event, B for beliefs or thoughts, and C for emotional outcomes), and relaxation.	The mother was asked to draw negative thoughts, describe time, place, and trigger in the situation section, and write negative thoughts in the next column and negative emotions in the column after. The downward arrow method helped the mother to achieve the basic schema, and then the technique of challenging negative thoughts was used. Types of cognitive errors and classification of automatic thoughts were explained.
Third session	The pregnant woman was given the opportunity to become acquainted with important cognitive distortions in order to identify these intellectual errors in her thoughts and examine them using the downward arrow method. Mindfulness training, strengthening positive thoughts, and relaxation methods were carried out.	Participants were asked to describe their obsessive-compulsive behaviors. Then the reinforcement of the mother’s positive thoughts and the consequences of beliefs and practice of thought induction were explained. Homework was provided.
Fourth session	An overview of the previous session, plan to change automatic thoughts, and train mindfulness were done, the woman was encouraged to continue the exercises and give feedback.	Mindfulness training and empowerment of participants to have mindfulness in daily activities were performed.

### Instrument and outcomes

The instrument used in the present study had two parts: The first part was related to demographic characteristics, including maternal age, body mass index, education status, employment status, economic status, ethnicity, history of abortion, gestational age at the time of enrollment, and recent gestational status. The second part was the Spielberger State-Trait Anxiety Inventory (STAI).

The State-Trait Anxiety Inventory (STAI) can be used to diagnose and differentiate between depressive syndromes. The Spielberger state-trait inventory has 40 self-reporting items that measure state anxiety (first 20 items) and trait anxiety (second 20 items). The scoring system in this inventory is based on the Likert scale ranging from 1 (very low) to 4 (very high). This scale has good validity and reliability. The items that indicate no anxiety are scored in reverse. For items 1 to 20 in each of the state and trait sections, a minimum of 20 to a maximum of 80 scores is considered. Classification of state anxiety includes mild (20–31), moderate to low (32–42), moderate to high (43–53), moderate to severe (54–64), severe (65–75), and very severe (76 and above). Also, classification of trait anxiety includes mild (20–31), moderate to low (32–42), moderate to high (43–52), relatively severe (53–62), severe (63–72), and very severe (73 and above), [30]. The validity and reliability of the Persian version of this tool have been confirmed by Mahram [33].

In this study, the STAI questionnaire was completed before and immediately after the intervention (before obtaining the amniocentesis result) in the intervention and control groups, and scores were compared between the two groups.

### Sample size

To determine the minimum sample size at 95% confidence level and 80% test power, assuming that the effect of training on state anxiety of pregnant women with positive screening results for chromosomal abnormalities in the intervention group should be 5 units more than the control group (average 10% of instrument score) to be considered statistically significant, the minimum sample size required in each group was calculated to be 35 people using the following formula:

$${\displaystyle \begin{array}{c}n=\frac{{\left({z}_{1-\alpha\left/2\right.}+{z}_{1-\beta}\right)}^2\times \left({s}_1^2+{s}_2^2\right)}{d^2}\\ {}{z}_{0.975}=1.96\\ {}{z}_{0.8}=0.84\\ {}\mathrm{d}=5\\ {}{s}_1=7.2\\ {}{s}_2=7.6\\ {}n=\frac{{\left(1.96+0.84\right)}^2\times \left({7.2}^2+{7.6}^2\right)}{5^2}=35\end{array}}$$

However, 42 people were assigned to each group by taking into account 20% sample drop. Since the standard deviation of state anxiety was higher than the trait anxiety, a higher sample size was considered with the help of this variable, which also covered the other research variables such as trait anxiety [34].

### Statistical analysis

Data were analyzed by SPSS software version 22 using descriptive and inferential statistics. Descriptive statistics such as numerical indexes and frequency distribution tables were used to describe the data. Chi-square test and Fisher’s exact test were used to compare the characteristics of the two groups for qualitative variables and independent t-test and ANOVA were used for quantitative variables (Based on Kolmogorov-Smirnov test, all of the quantitative variables had normal distribution). Also, within-group comparison was performed by paired t-test, and efficacy was evaluated by the analyses of covariance (ANCOVA), which is an extension of ANOVA that allows assessment of group differences in terms of dependent variable after controlling the effect of other covariates (e.g., time 1 variables). Clinical significance was estimated using the partial eta square effect size from ANCOVA, which represents variance explained by the cognitive-behavioral training vs. control group, after eliminating the effect of covariates. According to per Colin et al., partial eta square effect sizes are classified as small (0.01), medium (0.06), and large (0.14 and higher), [35]. The effect sizes in within groups comparisons were reported based on Cohen’s d, and Standardized Mean Difference was reported based on Cohen’s d effect size (null effect = 0, trivial effect = 0 – 0.19, small effect = 0.2 = 0.49, medium effect = 0.5 – 0.79, large effect = 0.8 – 1.19, very large effect = 1.2 – 2, and huge effect ≥2), [36, 37]. It should be noted that the analysis process of the current study was performed by using both intention to treat and per-protocol approaches. Also, the missing state and trait anxiety values were imputed with a multiple imputation model [38]. The significance level of less than 0.05 was considered. For multiple tests, the *p*-value was adjusted based on Bonferroni correction.

## Results

### Samples’ characteristics

A total of 92 samples were included in the study. In the intervention group, 4 people were excluded (2 due to abortion during the study and 2 due to participating in only one session). Also in the control group, 4 people were excluded (3 due to abortion during the study and 1 for unwillingness to continue with the study). The analysis in the current study was performed by both intention to treat and per-protocol approaches on 92 and 84 participants, respectively (Fig. 1). According to the findings, there was no statistically significant difference between the control and intervention groups in terms of individual variables (Table 2).

**Table 2:** Participants’ characteristics and tests used to compare pre-trial differences between the two groups

Variables	Cognitive- behavioral educations group (***n*** = 46)	Control group (***n*** = 46)	^a^*P* value
**Maternal age (year),** mean (SD^b^)	28.8 (5.42)	28.2 (6.7)	0.623
**BMI**, mean (SD)	24.73 (4.06)	24.67 (3.72)	0.952
**Gestational age at beginning of study**, mean (SD)	12.36 (0.91)	12.44 (1.2)	0.081
**Education status**, n (%)			0.474
Diploma	14 (30.43)	21 (45.65)	
Bachelor’s degree and higher	32 (69.57)	25 (54.35)	
**Occupational status**, n (%)			0.062
Housewife	26 (56.6)	34 (73.9)	
Employed	20 (43.5)	12 (26.1)	
**Economic status**, n (%)			0.506
Undesirable	18 (39.2)	17 (37)	
Relatively desirable	22 (47.8)	26 (56.5)	
Desirable	6 (13)	3 (6.5)	
**Ethnicity**, n (%)			0.076
Lor	4 (8.7)	10 (21.7)	
Fars	27 (58.7)	19 (41.3)	
Turk	7 (15.2)	9 (19.6)	
Kurd	7 (15.2)	3 (6.5)	
Other	1 (2.2)	5 (10.9)	
**Pregnancy status**, n (%)			0.205
Wanted	25 (54.35)	24 (52.17)	
Unwanted	21 (45.65)	22 (47.83)	

^a^*P* <  0.05 is significant

^b^Standard deviation

### Intervention’s effects on anxiety

According to Table 2, there was statistically significant difference in the mean scores of state anxiety between the two groups before the intervention. There was also no statistically significant difference in the mean scores of trait anxiety between the two groups before the intervention. However, after the intervention, there was statistically significant difference in state anxiety scores (*p* <  0.001) and trait anxiety scores (*p* <  0.001) between the intervention and control groups once their means were adjusted for pre-intervention scores by using the ANCOVA test. The results showed a large effect size between the groups in terms of both state and trait anxiety scores clinically based on partial eta square. The intervention group showed a statistically significant and a large decrease in state and trait anxiety scores from pre-trial to post-trial. In contrast, the control group showed a statistically significant and medium increase in state and trait anxiety scores from pre-trial to post-trial. The differential pattern of change for the intervention and control groups is illustrated separately in Tables 3 and 4 based on intention-to-treat and per-protocol analysis.

**Table 3:** Effect of intervention on outcomes - intention-to-treat sample

Variables	Cognitive- behavioral educations group (***n*** = 46)		Control group (***n*** = 46)		MD^**e**^ (CI^**f**^ 95%)	ES^**g**^ (Between)	***P***-value (Between groups)
	Mean (SD^a^)	ES^h^ (within)	Mean (SD^a^)	ES^h^ (within)			
**State anxiety**							
^b^Pre intervention	63.63 (6.13)	–	59.83 (7.42)	–	3.804 (0.98 to 6.62)	–	**0.009**
^c^Post intervention	40.2 (11.05)	2	65.52 (11.12)	0.59	−27.55 (−32.03 to −23.07)	0.63	**< 0.001**
^d^*P*-value (within groups)	< 0.001		< 0.001				
**Trait anxiety**							
^b^Pre intervention	58 (9.78)	–	56.2 (10.96)	–	1.804 (− 2.5 to 6.107)	–	0.407
^c^Post intervention	37.34 (8.44)	2.018	63.81 (11.28)	0.65	−27.33 (− 30.93 to - 23.73 to)	0.72	**< 0.001**
^d^*P*-value (within groups)	< 0.001		< 0.001				

^a^Standard Deviation

^b^Independent-samples t-test

^c^ANCOVA

^d^Paired-sample T Test

^e^Mean Difference

^f^Confidence Interval

^g^Effect size (ES) based on partial eta square

^h^Effect sizes based on Cohen’s d

**Table 4:** Effect of intervention on outcomes – per-protocol sample

Variables	Cognitive- behavioral educations group (***n*** = 46)		Control group (***n*** = 46)		MD^**e**^ (CI^**f**^ 95%)	ES^**g**^ (Between)	***P***-value (Between groups)
	Mean (SD ^a^)	ES ^h^ (within)	Mean (SD ^a^)	ES ^h^ (within)			
**State anxiety**							
^b^Pre intervention	63.88 (6.34)	–	59.90 (7.63)	–	3.98 (0.93 to7.02)	–	**0.011**
^c^Post intervention	38.21 (9.35)	2.68	66.14 (11.35)	0.63	−30.44 (− 34.72 to − 26.16)	0.71	**< 0.001**
^d^*P*-value (within groups)	< 0.001		< 0.001				
**Trait anxiety**							
^b^Pre intervention	57.76 (10.09)	–	55.71 (11.12)	–	2.095 (− 2.56 to 6. 75)	–	0.374
^c^Post intervention	36.02 (7. 5)	2.17	63.97 (11.76)	0.69	−28.96 (− 32.65 to - 25.27)	0.75	**< 0.001**
^d^*P*-value (within groups)	< 0.001		< 0.001				

^a^Standard Deviation

^b^Independent-samples t-test

^c^ANCOVA

^d^Paired-sample T Test

^e^Mean Difference

^f^Confidence Interval

^g^Effect size (ES) based on partial eta square

^h^Effect sizes based on Cohen’s d

## Discussion

The results of the current study showed a statistically and clinically significant difference in the state and trait anxiety scores of the cognitive-behavioral training group after the intervention compared to the control group. Also, changes in state and trait anxiety scores were positive in the control group, so that anxiety scores had increased after the study, but in the intervention group, these changes were negative. In other words, state and trait anxiety scores decreased after the intervention in the cognitive-behavioral training group.

An individual intervention based on cognitive-behavioral therapy in the Surkan et al., study decreased the anxiety and depression scores of pregnant women in low-income countries [39]. Consistent with the present study, Uguz et al.’s results showed that the level of anxiety symptoms after cognitive-behavioral therapy was significantly lower than the baseline level, so CBT seems to be a safe and effective treatment for anxiety disorder during pregnancy [40]. In another study, an internet-based cognitive-behavioral therapy reduced pregnancy anxiety and participants reported that iCBT was an acceptable treatment for prenatal anxiety [41].

The results of Salehi et al. (2016) study showed a significant reduction in the level of state and trait anxiety in CBT and interactive lecture groups. Also, the effect of group cognitive-behavioral therapy (GCBT) on reducing participants’ anxiety was greater than interactive lectures, but the difference was not significant [42]. Cognitive-behavioral therapy improved anxiety and related symptoms in women with anxiety disorders in the prenatal period in two other studies [25, 43].

In the study of Dayhimi et al. (2020), a significant difference was observed between the two groups of midwifery counseling and control after intervention in terms of state anxiety. However, there was no significant difference in trait anxiety. The content of midwifery counseling sessions consisted of two parts; physical and psychological contents. Physical content included physiology of pregnancy, the importance of nutrition, personal hygiene, oral health, sexual health, the symptoms of labor, and benefits of vaginal childbirth, breastfeeding training, post-partum birth control methods, and the essential tips for infant care. Psychological contents included the nature of pregnancy anxiety and its effects on mother and fetus, identifying negative thoughts and how to respond to them, how to control anxiety, and relaxation exercises that help to create a positive mental image [44]. The results of this study are consistent with the current study in terms of reducing state anxiety, but not in terms of trait anxiety. Trait anxiety is a personality trait that reflects the frequency and severity of emotional response to stress and in fact, this anxiety is a characteristic of the person and has nothing to do with the characteristics of the situation that the person is facing. The effect of intervention on trait anxiety refers to cognitive treatment techniques that teach a person to recognize and change dysfunctional beliefs and cognitive errors, and this can even reduce the recurrence of anxiety [45]. However, it seems that the educational content of psychological training in Dayhimi et al’ study has paid less attention to the correction of dysfunctional beliefs and cognitive errors, while the content of physical training has been more prominent.

In another study, an 8-week mindfulness-based cognitive therapy intervention in pregnant women was able to reduce the symptoms of depression and anxiety. In this study, the mothers were trained to gain more awareness about their thoughts, feelings, and body emotions, and establish a different relationship with them [46]. The results of this study are consistent with the findings of the current study. In the study of Darrehshouri-Mohammadi et al. (2013), the findings showed that stress management training decreased the state/trait anxiety and pregnancy anxiety. Therefore, they recommended cognitive-behavioral stress management training program during pregnancy as a suitable approach to promote mental health and reduce pregnancy stress and anxiety in primiparous women [47].

Numerous studies show that CBT is more effective in treating anxiety and depression. Cognitive-behavioral therapy is one of the psychotherapy approaches that aim to help people overcome emotional problems [48]. This treatment causes fundamental changes in a person’s beliefs, attitudes, feelings, thoughts, and emotions. Therefore, it can help clients in the intervention group to learn more adaptive ways to deal with anxiety, which ultimately leads to the development of a more adaptive way of thinking and behaving in dealing with anxious issues [49]. This intervention is especially preferred during pregnancy for mild to moderate anxiety disorders. Since in the first trimester of pregnancy, it is necessary to avoid any medicine as much as possible, this method can be especially useful during the 6-15 weeks of gestation, which is the time of fetal organogenesis [50].

In a study by Bittner et al. (2015), the cognitive-behavioral group therapy was able to make a significant difference in the level of anxiety between the intervention and control groups [51]. In this study, according to the inclusion criteria, people with high levels of anxiety were selected, and it seemed that cognitive-behavioral group therapy, as the only way to reduce anxiety levels in people with high levels of anxiety, was not effective and more interventions were required. These results are not in line with the findings of the current study. In another study, CBT was used to prevent prenatal depression and anxiety in low-income Central American immigrant women and results showed no significant difference between the two groups in terms of anxiety and depression [52], which is not consistent with the present study. To justify these different results, high-risk Latin women were selected who had under diploma education, which seems to be a complex issue in the cognitive-behavioral approach, and perhaps it would have been better to use more sessions with simpler content for this sample.

The study of Kordi et al. (2015) showed that after the interventions, there was a statistically significant difference in the mean scores of state and trait anxiety of pregnant women between the intervention and control groups. However, there was no statistically significant difference in the anxiety scores between the individual training and control groups, and also between individual training and group training groups [34]. The results of this study are not consistent with the finding of present study. Regarding the greater effect of group interventions compared to individual interventions, it can be said that most women are more satisfied with hearing common experiences in group meetings, and also hearing the feelings of others helps them to consider their feelings logically, and this reduces their anxiety [53].

### Study’s strengths and limitations, and also some suggestions for future research

The most important strength of the present study was the randomized controlled design, which made it possible to conclude that the anxiety changes were caused by the intervention and not merely by the passing of time. This interpretation is supported by the fact that incremental changes were also observed in the control group in terms of anxiety during the study. To our knowledge, this has not been demonstrated before when it comes to any form of psychological intervention for anxiety of pregnant women with positive screening results for chromosomal disorders. Other strengths of the present study were the low loss of follow-up rates and the well-validated measures of anxiety. One of the limitations of the present study was the different amount of social support that individuals received from family and friends, which could affect their level of anxiety. In the current study, we tried to distort this effect by randomly assigning people to two groups. According to the type of cognitive-behavioral intervention used in this study, only pregnant women with high school diploma were included in the study and pregnant women with less education were excluded. Therefore, caution should be taken in interpreting the results of the present study. It is suggested that in future research, the effect of cognitive-behavioral group training on stress, anxiety, and concerns of pregnant women with positive chromosomal disorder screening results would be investigated. Another limitation of the present study was its method of data collection that was based on self-reporting. In future studies, it is best to use psychometric instruments to measure the variables. Other limitations of the study included lack of blinding, which was not possible to implement in this study due to the nature of interventions, but the outcome assessor and statistician were not aware of the intervention content in the groups and allocation of individuals in the study groups.

## Conclusion and implications for practice

The current study showed that the state and trait anxiety of pregnant women with positive screening results for chromosomal disorders after cognitive-behavioral training was lower in the intervention group than the control group. Due to the effectiveness of CBT training, we recommended this method to be used for the reduction of anxiety in pregnant with a positive screening result.

## Supplementary Information

**Additional file 1.** CONSORT 2010 checklist of information to include when reporting a randomised trial*.

## Data Availability

The datasets generated and analyzed during the current study are not publicly available due to the confidentiality of information, but they can be available through the corresponding author on reasonable request.
